# Convergence of Plasmid‐Mediated *tmexCD‐toprJ* With Critical Resistance Genes Fuels Spread of Multidrug Resistant Bacteria

**DOI:** 10.1002/advs.77169

**Published:** 2026-08-20

**Authors:** Kaiwen Song, Meng Wang, Xingyu Wu, Meng Cai, Bowen Li, Qi Wang, Xiaodong Guan, Qi Ding, Ruobing Wang, Hui Wang

**Affiliations:** ^1^ Department of Clinical Laboratory Peking University People's Hospital; Beijing Key Laboratory of Innovative & Transformable Warning and Intervention Technologies for Drug‐Resistant Pathogens Beijing China; ^2^ Institute of Medical Technology Peking University Health Science Center Beijing China; ^3^ Department of Pharmacy Administration and Clinical Pharmacy School of Pharmaceutical Sciences Peking University Beijing China; ^4^ International Research Centre for Medicinal Administration Peking University Beijing China

**Keywords:** *bla*_KPC_/*bla*_NDM_/*mcr*, co‐resistance, epidemiology, *tmexCD‐toprJ*

## Abstract

The plasmid‐mediated tigecycline resistance cluster *tmexCD‐toprJ* is an emerging clinical threat whose convergence with carbapenemase and colistin resistance determinants may compromise multiple last‐line therapies. We analyzed 1227 *tmexCD‐toprJ*‐positive genomes from 42 countries and 16 genera, including 1189 public genomes and 38 newly sequenced clinical isolates. The increase was most pronounced in China, where *tmexCD‐toprJ* prevalence rose from 0.282% before 2011 to 1.504% after 2020, and approximately half of post‐2015 Chinese isolates were classified as dual‐positive, co‐harboring *bla*_NDM_, *bla*_KPC_, or *mcr*. *tmexCD‐toprJ* was mainly plasmid borne and distributed across diverse *Klebsiella* and *Pseudomonas* lineages and plasmid backbones. Dual‐positive plasmids showed distinct contexts: acquisition of *tmexCD‐toprJ* by pre‐existing *bla*_KPC_‐ or *mcr*‐positive IncF backbones, and remodeling of multiple resistance regions in *tmexCD‐toprJ*‐*bla*_NDM_ IncU/IncHI1B plasmids. Ecological distributions also differed; *bla*_NDM_ combinations spanned human, animal, and environmental sources, whereas *bla*_KPC_ and *mcr* combinations were mainly human and animal associated, respectively. Clinical isolates experiment confirmed resistance to the corresponding antibiotic classes, stable maintenance of dual‐positive plasmids, and transferability of a subset of plasmids. Under antibiotic pressure, the *tmexCD‐toprJ* region was mobilized across plasmids into a *bla*_KPC_ plasmid. These findings reveal multiple evolutionary routes driving convergence of last‐line resistance determinants and highlight the need for integrated genomic and phenotypic surveillance.

## Graphical abstract

Global dissemination of *tmexCD‐toprJ* across bacterial hosts and ecological niches is accompanied by its convergence with *bla*_KPC_, *bla*_NDM_, and *mcr* on mobile plasmids. Recombination and acquisition of resistance modules generate distinct multidrug resistant plasmid architectures, facilitating the emergence and spread of high‐risk bacterial populations across One Health settings.

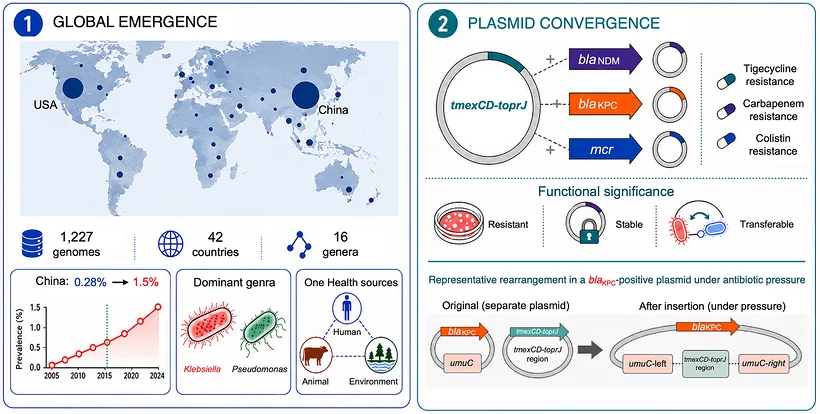

## 1 Introduction

The global expansion of multidrug resistant Gram‐negative bacteria has severely reduced the effectiveness of last‐line antibiotics, including tigecycline, carbapenems, and colistin. Tigecycline remains an important treatment option for infections caused by carbapenem resistant Enterobacterales and other multidrug resistant organisms. However, tigecycline resistance has increasingly been reported worldwide, raising concern that one of the remaining therapeutic options for severe infections may be compromised.

The plasmid‐mediated *tmexCD‐toprJ* gene cluster was first reported in *Klebsiella pneumoniae* from veterinary and clinical settings in China and represents an important mechanism of tigecycline resistance [1, 2]. Unlike classical tetracycline resistance determinants, *tmexCD‐toprJ* encodes a Resistance‐Nodulation‐Division (RND) family efflux pump that reduces susceptibility to tigecycline and structurally related antimicrobials [3]. Since its initial discovery, *tmexCD‐toprJ*‐positive isolates have been reported across multiple continents, including Asia, North and South America, Europe, and Oceania, indicating rapid international dissemination [4]. Its frequent localization on plasmids and association with mobile genetic elements immediately raised concern regarding its potential for horizontal dissemination across bacterial hosts.

At the same time, resistance to other last‐line antibiotics continues to increase [5, 6, 7]. Carbapenem resistance is frequently mediated by carbapenemase genes such as *bla*_KPC_ and *bla*_NDM_ [8, 9, 10], while colistin resistance can be mediated by mobilized colistin resistance genes such as *mcr* [10]. The co‐occurrence of *tmexCD‐toprJ* with *bla*_KPC_, *bla*_NDM_, or *mcr* is clinically concerning because it links resistance to tigecycline with resistance to carbapenems and/or colistins, thereby narrowing already limited treatment options for severe Gram‐negative bacterial infections [11, 12, 13].

Previous studies have shown that *tmexCD‐toprJ* can be carried by diverse plasmids, including IncU, IncF, IncHI, and other plasmid types [14], and can be associated with insertion sequences (IS), transposon‐like structures, and other mobile genetic elements [15, 16]. *TmexCD‐toprJ* has also been detected in multiple bacterial genera [17, 18, 19, 20] and in isolates from humans, animals, food production, and environmental sources, indicating its potential for cross‐niche dissemination under a One Health framework [21, 22, 23, 24]. However, most existing reports have focused on local outbreaks, individual plasmids, or specific bacterial hosts. The global genomic epidemiology of *tmexCD‐toprJ*, the plasmid contexts in which it converges with *bla*_KPC_, *bla*_NDM_, or *mcr*, and the phenotypic and functional relevance of such convergence remain incompletely understood.

A key unresolved question is whether the co‐occurrence of *tmexCD‐toprJ* with other critical resistance genes is merely a descriptive genomic phenomenon or whether it generates stable and transferable plasmid structures with clinical relevance. Another important issue is whether specific plasmid backgrounds or recombination associated regions may provide structural contexts for the form of dual‐positive plasmids. Addressing these questions requires integration of global genomic data with phenotypic and experimental validation.

In this study, we integrated large‐scale public genomic data with newly sequenced clinical isolates to define the global distribution, prevalence, host range, plasmid contexts, resistance gene combinations, and genetic environments of *tmexCD‐toprJ*. We further performed antimicrobial susceptibility testing, plasmid stability assays, conjugation experiments, competition assays, and an antibiotic‐pressure plasmid convergence experiment to assess the functional relevance of representative dual‐positive plasmids. Together, these analyses show that *tmexCD‐toprJ* can converge with *bla*_KPC_, *bla*_NDM_, and *mcr* in distinct plasmid backgrounds, generating stable, phenotypically resistant, and in some cases transferable high‐risk resistance plasmids.

## 2 Results

### 2.1 Global Distribution of *tmexCD‐toprJ*‐Positive Isolates and Rising Co‐Resistance in China

To define the global genomic landscape of *tmexCD‐toprJ*‐positive bacteria, we screened the NCBI Pathogen Detection database, which included 1 735 923 global bacterial pathogen isolates (June 2024), and 1189 non‐redundant public *tmexCD‐toprJ‐*positive genomes were retained. In parallel, 38 *tmexCD‐toprJ*‐positive clinical isolates were newly sequenced from multicenter surveillance in China. Together, these formed a final dataset of 1227 non‐redundant *tmexCD‐toprJ*‐positive genomes.

**FIGURE 1:**
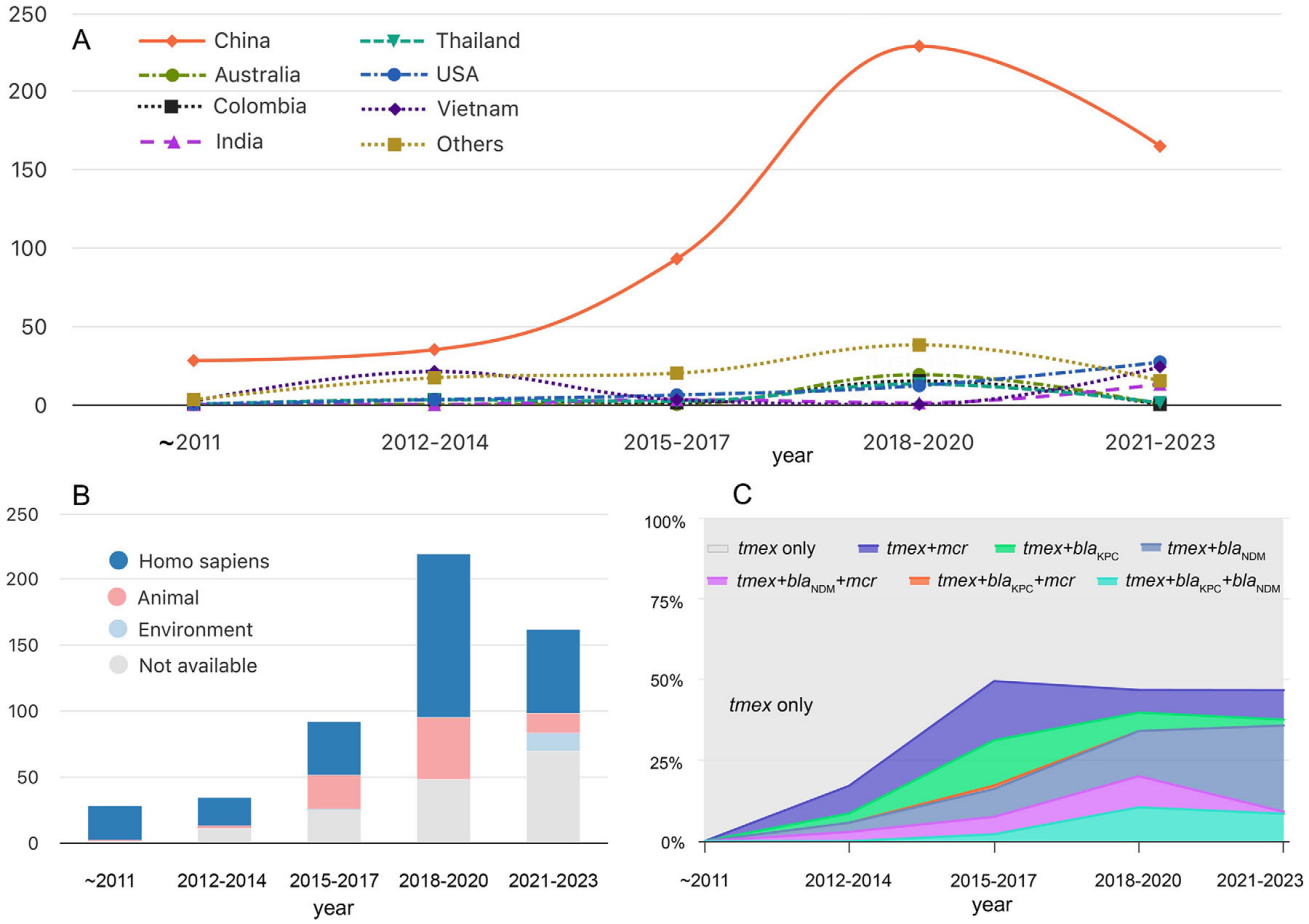
Global and temporal distribution of *tmexCD‐toprJ*‐positive isolates and their genetic contexts (A)Temporal trends in the number of *tmexCD‐toprJ*‐positive isolates reported across different countries (B) Source types of *tmexCD‐toprJ*‐positive isolates over time in China, categorized into humans, animals, environment, and not available. (C) Temporal distribution of co‐occurring resistance determinants in *tmexCD‐toprJ*‐positive isolates in China. For simplicity, *tmex* denotes *tmexCD‐toprJ* in the figure.

*TmexCD‐toprJ*‐positive isolates were identified in 42 countries and 16 bacterial genera, showing a broad but uneven global distribution (Figure S1A). The largest number of genomes was detected in China, followed by the United States and Vietnam (Figure 1A). Among 1 401 543 isolates with available collection year and geographic metadata, 769 were *tmexCD‐toprJ*‐positive and were used for prevalence estimation. This analysis showed a post‐2015 increase, particularly in China, where prevalence rose from 0.282% before 2011 to 1.504% after 2020 (Figure S2). In China, early *tmexCD‐toprJ*‐positive samples were almost exclusively from human sources. Over time, however, an increasing proportion of isolates have been recovered from animals and the environment, with the human source proportion shrinking, except during 2021–2023, when COVID‐19 pandemic related sampling constraints limited non‐human sampling (Figure 1B).

After 2015, approximately half of Chinese *tmexCD‐toprJ*‐positive isolates co‐harbored at least one critical resistance determinant, including *bla*_NDM_, *bla*_KPC_, or *mcr* (Figure 1C). These isolates were hereafter defined as dual‐positive isolates. Among these combinations, *tmexCD‐toprJ*‐*bla*_NDM_ was the most frequent and was increasingly detected in both co‐plasmid and co‐strain contexts (Figure S1C), and *Klebsiella* accounted for the majority of dual‐positive isolates (Figure S1D), highlighting the need to monitor *tmexCD‐toprJ* together with co‐carried carbapenemase and colistin resistance determinants.

### 2.2 Genus Specific Distribution and Plasmid‐Mediated Dissemination of *tmexCD‐toprJ*‐Positive Isolates

**FIGURE 2:**
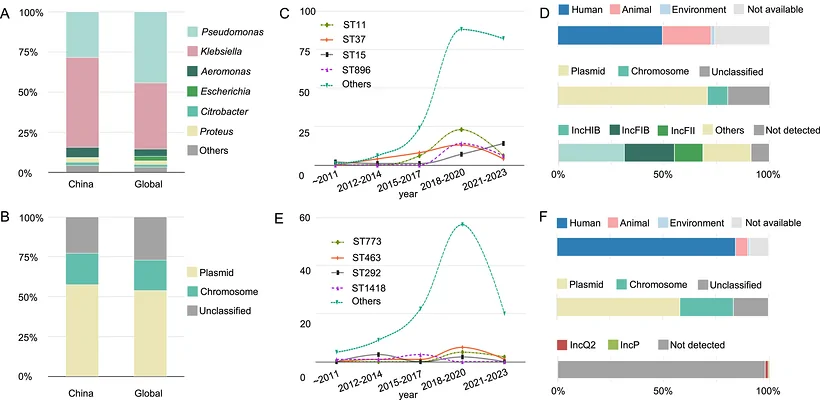
Distribution and characteristics of *tmexCD‐toprJ*‐positive isolates in China. (A) Genus level distribution of tmexCD‐toprJ‐positive isolates in China and globally. (B) Genomic location of *tmexCD‐toprJ* genes among isolates in China and globally. (C) Temporal trends of MLST carrying *tmexCD‐toprJ* in *Klebsiella*. (D) Source types, genomic location, and plasmid replicon types of *tmexCD‐toprJ* in *Klebsiella*. (E) Temporal trends of MLST *tmexCD‐toprJ*‐positive isolates in *Pseudomonas*. (F) Source types, genomic location, and plasmid replicon types of *tmexCD‐toprJ* in *Pseudomonas*.

The genus distribution of *tmexCD‐toprJ*‐positive isolates was dominated by *Klebsiella* and *Pseudomonas*, which together accounted for over 80% of the dataset (Figure 2A). *Pseudomonas* has been proposed as an important reservoir of novel *tmexCD‐toprJ* variants, but genus composition varied markedly by region [25]. In China, *Klebsiella* predominated among both *tmexCD‐toprJ*‐only isolates and dual‐positive isolates, accounting for 47.8% and 77.0% of these groups, respectively. In contrast, *Pseudomonas* accounted for 93.8% of dual‐positive isolates in the United States.

The relative contribution of *Klebsiella* and *Pseudomonas* also changed over time in China. Before 2011, *Pseudomonas* represented 89% of *tmexCD‐toprJ*‐positive isolates. From 2012 onwards, *Klebsiella* gradually increased in prevalence, rising from 31% in 2012–2014 to 67% in 2021–2023, while *Pseudomonas* declined from 40% to 14% over the same period. This temporal transition coincided with the post‐2015 rise in Chinese dual‐positive isolates. Phylogenetic reconstruction of *tmexCD‐toprJ*‐positive plasmids revealed that plasmids co‐harboring *tmexCD‐toprJ* with *bla*_NDM_, *bla*_KPC_, or *mcr* each formed closely related phylogenetic clusters, indicating that plasmids carrying the same resistance gene combination share highly similar genetic backgrounds. The close phylogenetic relationship between *tmexCD‐toprJ*‐only plasmids and dual‐positive plasmids further suggested that these plasmids may share related backbone structures (Figure S3A).

To assess whether *tmexCD‐toprJ* dissemination was mainly associated with clonal expansion, we analyzed bacterial Multi‐locus sequence typing (MLST) in the major host species. In *Klebsiella*, *tmexCD‐toprJ* occurred across diverse MLSTs, including ST11, ST37, ST15, ST1, ST896, and less common STs. In *Pseudomonas*, *tmexCD‐toprJ* also occurred across multiple MLSTs, including ST773, ST463, ST292, ST1418, and others (Figure 2C,E). This broad bacterial ST distribution, with no predominant ST, argues against spread by a single dominant bacterial clone. Consistently, more than half of *tmexCD‐toprJ* loci were plasmid borne (Figure 2B), and structural analysis of the surrounding genetic contexts identified six predominant mobilization architectures, indicating multiple potential routes of horizontal transfer (Figure S3B). Together, the broad MLST distribution, plasmid localization, and diverse mobilization structures are more consistent with repeated horizontal acquisition and plasmid‐mediated dissemination than with purely clonal spread.

Ecological and plasmid distributions further differed between the two dominant genera. In China, *Klebsiella* isolates were mainly recovered from human and animal sources, with IncHIB plasmids predominating, followed by IncFIB and IncFII plasmids. By contrast, *Pseudomonas* isolates were primarily human derived, with approximately 90% recovered from clinical settings, and were also mainly associated with IncHIB plasmids (Figure 2D,F).

### 2.3 Genomic Features Associated With Co‐Carriage of *tmexCD‐toprJ* and Critical Resistance Genes

**FIGURE 3:**
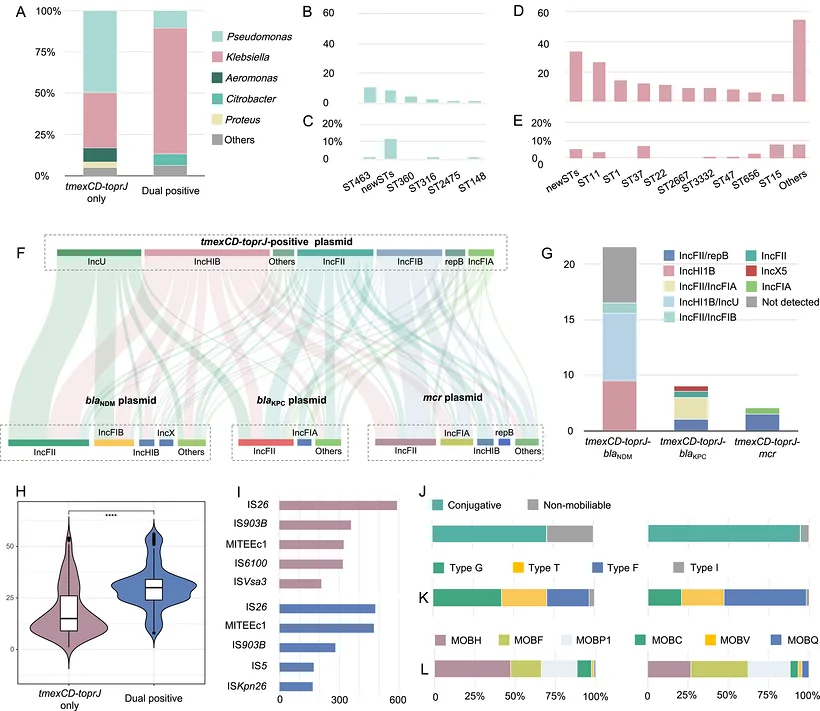
Distribution, MLSTs, and plasmid replicon types of dual‐positive isolates. (A) Genus composition of *tmexCD‐toprJ*‐only and dual‐positive isolates. (B) Distribution of sequence types among dual‐positive *Pseudomonas* isolates. (C) Proportion of *Pseudomonas* STs in *tmexCD‐toprJ*‐only isolates, corresponding to STs shown in panel B. (D) Distribution of STs among dual‐positive *Klebsiella* isolates. (E) Proportion of *Klebsiella* STs in *tmexCD‐toprJ‐*only isolates, corresponding to STs shown in panel D. (F) Cooccurrence network of plasmid types associated with *tmexCD‐toprJ*, *bla*_NDM_, *bla*_KPC_, and *mcr* plasmids in dual‐positive isolates. (G) Replicon types of dual‐positive plasmids. (H) Number of IS per genome in *tmexCD‐toprJ*‐only and dual‐positive strains. (I) Top five most abundant IS elements identified in *tmexCD‐toprJ*‐only and dual‐positive strains. (J) Proportion of strains harboring conjugation modules in *tmexCD‐toprJ*‐only (left) and dual‐positive strains (right). (K) Distribution of type IV secretion system (T4SS) types among strains with conjugative modules. (L) Distribution of relaxase types among strains with conjugative modules.

To characterize genomic features associated with isolates co‐harboring *bla*_NDM_, *bla*_KPC_, or *mcr*, we compared *tmexCD‐toprJ*‐only isolates and dual‐positive isolates. The two groups showed distinct genus compositions. Among *tmexCD‐toprJ*‐only strains, *Pseudomonas* accounted for 50% of isolates, whereas *Klebsiella* represented over 70% of dual‐positive isolates (Figure 3A).

Bacterial MLST distributions also differed between the two groups in the two major host species. In dual‐positive *Pseudomonas* isolates, ST463 was the most prevalent sequence type, whereas *tmexCD‐toprJ*‐only *Pseudomonas* isolates were dominated by newly emerging STs (Figure 3B,C). In *Klebsiella*, dual‐positive isolates were mainly represented by newSTs, ST1 and ST11, while *tmexCD‐toprJ*‐only isolates were enriched for ST37 and ST15 (Figure 3D,E). All environmental or animal derived dual‐positive ST1 isolates recovered between 2017 and 2019 carried *mcr* gene. By contrast, ST11 isolates were detected across human, animal, and environmental sources. In human isolates, ST11 isolates more often co‐harbored *bla*_NDM_ or *bla*_KPC_, whereas animal or environmental‐associated isolates more frequently co‐harbored *mcr*.

We next compared mobile genetic elements and conjugation associated features between *tmexCD‐toprJ*‐only and dual‐positive isolates. Dual‐positive isolates carried significantly more insertion sequences than *tmexCD‐toprJ*‐only isolates, with a median of 30 IS per genome compared with 15 in *tmexCD‐toprJ*‐only strains (Figure 3H, *p* <0.001). Among these elements, IS*26*, IS*903B*, and MITEEc1 (miniature inverted‐repeat transposable element) were the three most prevalent (Figure 3I). Dual‐positive isolates also show a higher proportion of plasmids carrying conjugation‐related modules than *tmexCD‐toprJ*‐only isolates (92.1% VS 72.7%, Figure 3J). Type IV secretion system (T4SS) and relaxase profiles further differed between the two groups: dual‐positive strains predominantly harbored TypeF T4SS and MOBF/MOBP1 relaxases, whereas *tmexCD‐toprJ*‐only strains showed higher frequencies of Type G T4SS and MOBH relaxases (Figure 3K,L).

In addition, *tmexCD‐toprJ*‐positive plasmids also encoded diverse anti‐defense modules. Over 60% of these plasmids carried at least one anti‐defense gene, and their anti‐defense repertoire diversity, measured by Shannon entropy, was significantly higher than that of other plasmid groups (Figure S4A,B). These modules may help plasmid overcome host defense barriers, such as restriction‐modification or CRISPR‐Cas systems.

### 2.4 Structural Features and Co‐Localization Patterns of *tmexCD‐toprJ‐*Positive Plasmids

Plasmid characterization showed *tmexCD‐toprJ*‐positive plasmids predominantly within the 100–400 kb range (Figure S5). Composite structures represented a substantial proportion, with IncU, repB, IncF, and IncH as predominant replicon types, alongside diverse accessory replicons (Figure S5). Among these major plasmids, they also exhibit a strong capacity to carry other resistance genes. Analysis of IncU (Figure S6) and repB (Figure S7) plasmids, the primary *tmexCD‐toprJ*‐positive types from public databases, revealed these plasmids to be versatile, as they can also harbor common resistance genes such as *bla*_NDM_, *bla*_KPC_, and *mcr*, with over 20% carrying at least one of these genes. Resistance gene co‐occurrence analysis demonstrated different replicon typability in plasmids co‐harboring *tmexCD‐toprJ* with *bla*_NDM_ or *bla*_KPC_. Specifically, *tmexCD‐toprJ*‐*bla*_NDM_ co‐localization showed preferential association with IncU and IncHIB replicons, whereas *tmexCD‐toprJ*‐*bla*_KPC_/*mcr* combinations linked predominantly to IncF replicons. In total, 59 dual‐positive plasmids were identified (Table S2).

At the strain level, analysis of dual‐positive isolates revealed distinct co‐occurrence patterns between *tmexCD‐toprJ* and *bla*_NDM_, *bla*_KPC_, and *mcr* genes, as shown in the plasmid type network (Figure 3F). In *tmexCD‐toprJ*‐*bla*_NDM_‐positive isolates, *tmexCD‐toprJ* was primarily localized on IncU or IncHIB plasmids, while *bla*_NDM_ was most frequently associated with IncFII or IncFIB plasmids. In *tmexCD‐toprJ*‐*bla*_KPC_positive isolates, *tmexCD‐toprJ* was carried on IncU, IncHIB, or IncFII plasmids, in combination with *bla*_KPC_ located on IncFII plasmids. For *tmexCD‐toprJ*‐*mcr* positive isolates, *tmexCD‐toprJ* was mainly found on IncHIB and IncFIB plasmids, whereas *mcr* was linked to IncFII and IncFIA plasmids. Consistent with these strain‐level associations, replicon typing of dual‐positive plasmids (Figure 3G) further delineated the predominant plasmid backbones involved. *tmexCD‐toprJ*‐*bla*_NDM_ plasmids were mainly classified as IncHI1B/IncU and IncHI1B types, *tmexCD‐toprJ*‐*bla*_KPC_ plasmids were dominated by IncFII and IncFIA replicons, and *tmexCD‐toprJ*‐*mcr* plasmids were primarily associated with IncFII and repB replicons.

**FIGURE 4:**
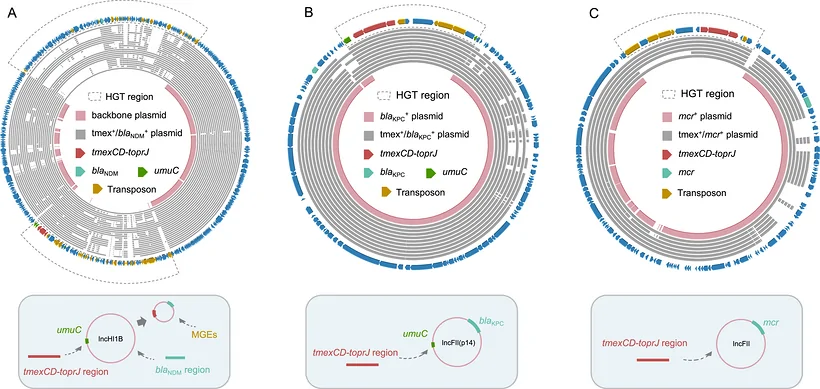
Formation processes of dual‐positive plasmids. In the circular diagrams: The innermost circle represents the background reference strain. Each outer gray circle represents the mapping from a dual‐positive plasmid to the reference strain. The outermost circle shows the gene annotation for the background strain. (A) *tmexCD‐toprJ‐bla*_NDM_: The two resistance genes (*tmexCD‐toprJ* and *bla*_NDM_) are inserted into a new IncHI1B plasmid. (B) *tmexCD‐toprJ‐bla*_KPC_: The *tmexCD‐toprJ* gene cluster is inserted into the plasmid harboring *bla*_KPC_. (C) *tmexCD‐toprJ‐mcr*: The *tmexCD‐toprJ* gene cluster is inserted into the plasmid harboring *mcr*.

To better understand the structural dynamics underlying dual‐positive plasmids, we compared plasmids co‐harboring *tmexCD‐toprJ* and *bla*_KPC_, *bla*_NDM_, or *mcr* with related sequences in the NCBI database. This comparison allowed us to trace possible backbone structures and infer precursor forms. For IncF‐based *tmexCD‐toprJ*‐*bla*_KPC_ and *tmexCD‐toprJ*‐*mcr* plasmids, related backbone sequences already contained intact *bla*_KPC_ or *mcr* regions, whereas the *tmexCD‐toprJ* region was associated with a putative mobile element, suggesting possible acquisition of *tmexCD‐toprJ* by pre‐existing *bla*_KPC_‐ or *mcr*‐positive plasmid backbones (Figure 4B,C). In contrast, *tmexCD‐toprJ‐bla*_NDM_ plasmids showed a more complex pattern (Figure 4A): related backbone sequences lacked both *tmexCD‐toprJ* and *bla*_NDM_, while the two resistance regions were each associated with putative mobile elements and displayed substantial recombination diversity. These observations suggest that the assembly of *tmexCD‐toprJ‐bla*_NDM_ plasmids may involve independent acquisition and remodeling of multiple resistance regions rather than a single simple insertion event. Overall, these findings indicate that dual‐positive plasmids can form through multiple structural routes, with *tmexCD‐toprJ* converging with clinically important resistance determinants through mobile element associated recombination and plasmid backbone remodeling.

### 2.5 Phenotypic Relevance, Stability, and Transferability of Dual‐Positive Plasmids

To determine whether genomic co‐carriage corresponded to resistance phenotype and functional plasmid behavior, we analyzed the 38 in‐house strains. These included 8 strains carrying only *tmexCD‐toprJ*‐positive plasmids, 15 strains in which *tmexCD‐toprJ* and *bla*_KPC_, *bla*_NDM_, or *mcr* were located on the same plasmid, and 15 strains in which *tmexCD‐toprJ* and *bla*_KPC_, *bla*_NDM_, or *mcr* coexisted in the same isolate but were located on different plasmids (Table S2).

**FIGURE 5:**
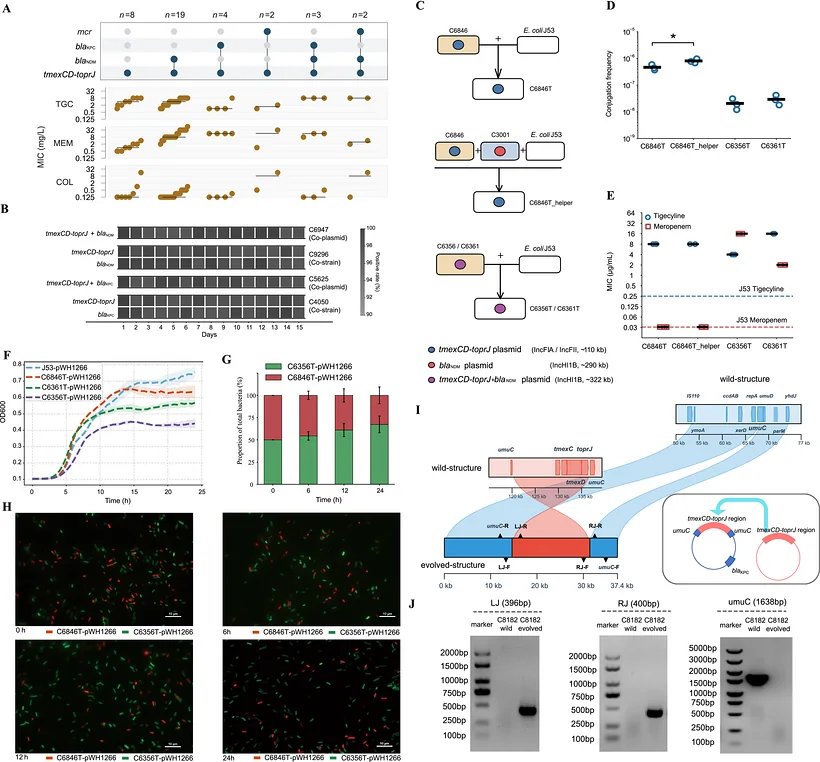
Phenotypic resistance, plasmid stability, transferability, and antibiotic pressure associated plasmid rearrangement in *tmexCD‐toprJ*‐positive isolates. (A) Antimicrobial susceptibility profiles of *tmexCD‐toprJ*‐positive isolates grouped by plasmid configuration: *tmexCD‐toprJ*‐only, dual‐positive co‐strain carriage, or dual‐positive co‐plasmid carriage with *bla*_NDM_, *bla*_KPC_, or *mcr*. Minimum inhibitory concentrations (MICs) are shown for tigecycline, meropenem, and colistin. (B) Stability of *tmexCD‐toprJ* and co‐carried carbapenemase genes during 15‐day serial passage without antibiotic selection. Four representative isolates were monitored, including co‐plasmid and co‐strain configurations involving *bla*_NDM_ or *bla*_KPC_. (C) Schematic of conjugation assays using sodium azide resistant *E. coli* J53 as the recipient. Donors included the *tmexCD‐toprJ*‐only strain C6846 and dual‐positive strains C6356 and C6361; C3001 was used as the *bla*_NDM_‐positive helper strain. (D) Conjugation frequencies of *tmexCD‐toprJ*‐positive plasmids. ^*^, *p* <0.05. (E) Antimicrobial susceptibility profiles of transconjugants. Dashed lines indicate baseline MICs of the recipient strain J53 for tigecycline and meropenem. (F) Growth curves of J53 and transconjugants C6846T, C6356T, and C6361T in lysogeny broth. Data represent mean ± standard deviation from three biological replicates. (G) Competition assay between C6846T‐mCherry and C6356T‐GFP under 0.38 µg/mL tigecycline. Relative abundance was determined by colony‐forming unit enumeration at 0, 6, 12, and 24 h. (H) Fluorescence microscopy of competition co‐cultures. Green indicates C6356T‐GFP, and red indicates C6846T‐mCherry. Scale bar, 10 µm. (I) Structural model of antibiotic pressure associated *tmexCD‐toprJ* insertion into the *umuC*associated region of a *bla*_KPC_‐positive plasmid in evolved C8182. A ∼16.9‐kb *tmexCD‐toprJ* containing fragment was mobilized from its original plasmid into the *bla*_KPC_ plasmid. (J) PCR validation of left‐junction and right‐junction amplicons in parental and evolved C8182 isolates, confirming *umuC*‐associated plasmid rearrangement.

Antimicrobial susceptibility testing showed that dual‐positive isolates retained resistance to the corresponding antibiotics. Isolates carrying *tmexCD‐toprJ* showed reduced susceptibility or resistance to tigecycline, while isolates co‐harboring carbapenemase genes showed resistance to carbapenems, and isolates carrying *mcr* showed resistance to colistin or reduced susceptibility consistent with *mcr* carriage (Figure 5A). These results confirm that genomic co‐carriage of *tmexCD‐toprJ* with *bla*_KPC_, *bla*_NDM_, or *mcr* is phenotypically relevant in the representative isolates tested.

We next assessed plasmid stability. Dual‐positive strains were serially passaged without antibiotic selection, and plasmid retention was monitored by PCR and resistance gene screening. Dual‐positive plasmids were stably maintained after continuous passage, with no substantial loss of *tmexCD‐toprJ*, *bla*_KPC_, *bla*_NDM_, or *mcr* genes under the tested conditions (Figure 5B). The results indicate that high‐risk dual‐positive plasmids can persist even in the absence of direct antibiotic selection. Consistently, plasmid copy number (PCN) analysis of the newly sequenced genomes showed that *tmexCD‐toprJ‐*positive plasmids, including both *tmexCD‐toprJ‐*only and dual‐positive plasmids, did not show an obvious reduction in copy number despite their large size (Figure S11). This pattern deviates from the typical inverse relationship between plasmid size and copy number and may contribute to stable maintenance and sustained resistance gene dosage under non‐selective conditions.

Conjugation experiments were performed using sodium azide‐resistant *E. coli* J53 as the recipient (Figure 5C,D). Among 8 *tmexCD‐toprJ*‐only plasmid strains, strain C6846 yielded a *tmexCD‐toprJ*‐positive transconjugant at a frequency of 4.7 × 10^−7^ per recipient cell. 2 of 15 dual‐positive co‐plasmid strains yielded dual‐positive transconjugants: C6356 at 1.9 × 10^−8^ and C6361 at 2.8 × 10^−8^ per recipient cell. In contrast, none of the 15 strains in which *tmexCD‐toprJ* and *bla*_KPC_, *bla*_NDM_, or *mcr* were located on separate plasmids yielded detectable dual‐positive transconjugants under the same conditions. In addition, when the *bla*_NDM_‐positive strain C3001 was added to the C6846 conjugation system, the transfer frequency of the *tmexCD‐toprJ* plasmid increased from 4.7 × 10^−7^ to 8.1 × 10^−7^. Antimicrobial susceptibility testing of transconjugants confirmed that transferred plasmids retained or increased the corresponding resistance phenotypes (Figure 5E). These results do not imply that dual‐positive plasmids are more transmissible than *tmexCD‐toprJ‐*only plasmids. Rather, they show that some dual‐positive plasmids retain the capacity to transfer combined last‐line resistance determinants in a single horizontal transfer event, and suggest that the presence of *bla*_NDM_ positive bacteria may enhance the transfer potential of *tmexCD‐toprJ*‐positive plasmids under the tested conditions.

Finally, we compared the growth performance and competitive fitness of *tmexCD‐toprJ*‐only and dual‐positive transconjugants in the same *E. coli* J53 recipient background. Growth curve analysis showed that the *tmexCD‐toprJ*‐only transconjugant C6846T grew fastest, with a maximum growth rate of 0.491/h, followed by J53 (0.443/h), and the dual‐positive transconjugant C6361T (0.436/h) and C6356T (0.310/h) had the slowest growth rate (Figure 5F). Colony‐forming unit enumeration showed that the proportion of C6356T increased from approximately 50% at 0 h to approximately 68% at 24 h, while C6846T declined accordingly (Figure 5G). Fluorescence microscopy further confirmed progressive enrichment of GFP‐labeled C6356T cells in the mixed population (Figure 5H). Thus, although C6356T showed the lowest intrinsic growth rate in monoculture, it outcompeted the *tmexCD‐toprJ*‐only transconjugant under tigecycline exposure, indicating that dual‐positive plasmid carriage did not impose a prohibitive fitness cost and may provide a selective advantage under relevant antibiotic pressure.

### 2.6 *umuC*‐Associated Regions and Antibiotic Pressure Associated Plasmid Convergence

Comparative analysis of *tmexCD‐toprJ* genetic contexts revealed recurrent association with *umuC*‐containing plasmid regions. In our dataset, three dominant *umuC* clades showed distinct resistance gene associations: Clade I was mainly linked to *bla*_NDM_, Clade II to *bla*_KPC_, and Clade III to *bla*_NDM_ or *mcr*. These associations were further supported by screening more than 60 000 plasmids from PLSDB (Figure S8), suggesting that different *umuC* lineages may mark plasmid backgrounds associated with specific resistance gene combinations.

To experimentally assess the feasibility of *umuC*‐associated plasmid convergence, we performed an antibiotic‐pressure experiment using strain C8182, which carries *tmexCD‐toprJ* and *bla*_KPC_ on separate plasmids; the *bla*_KPC_ plasmid contains an intact Clade II *umuC* region. After five days of serial passage under increasing tigecycline and meropenem pressure, PCR analysis showed altered amplification patterns at the *umuC* locus in evolved isolates compared with the parental strain. Primers targeting the native *umuC* region amplified the parental strain but failed to amplify evolved isolates, indicating disruption of this region. Conversely, junction‐spanning primers designed from the predicted insertion structure produced no amplicon in the parental strain but yielded clear bands in evolved isolates (Figure 5I,J). Although this experiment does not demonstrate that all naturally occurring dual‐positive plasmids arise through the same route, it provides direct support for the feasibility of antibiotic pressure associated plasmid convergence through an *umuC*‐associated region in a representative plasmid background.

### 2.7 Ecological Distribution of Resistance Gene Combinations

We next examined the ecological distribution of dual‐positive isolates*. tmexCD‐toprJ‐bla*_NDM_ positive strains were detected across human, animal, and environmental sources, indicating broad cross‐niche distribution. In contrast, *tmexCD‐toprJ‐bla*_KPC_ positive strains were predominantly associated with human clinical sources, consistent with the clinical concentration of *bla*_KPC_‐producing pathogens. *tmexCD‐toprJ‐mcr* positive strains were more frequently associated with animal sources, consistent with the known association of *mcr* with food‐animal and agricultural reservoirs (Figure S9). Temporal metadata suggested that *tmexCD‐toprJ‐bla*_NDM_ positive isolates were detected earlier in human sources than in animal sources in the available dataset. Human derived detections were observed from 2013, whereas animal derived detections appeared later, with a peak after 2020. Because the dataset is subject to sampling bias and metadata incompleteness, we do not infer a definitive transmission direction. However, these patterns are consistent with ecological compartmentalization and possible cross‐niche movement of different resistance gene combinations.

**FIGURE 6:**
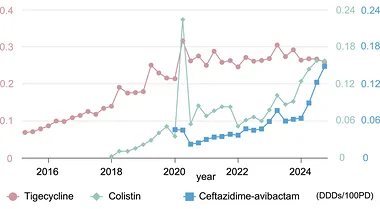
Trends in antimicrobial consumption in China in 2015–2024, expressed as Defined Daily Doses (DDD) per 100 patient‐days (PD).

We also compared regional antibiotic consumption trends. In China, increasing use of tigecycline, carbapenems, colistins, and ceftazidime‐avibactam coincided with the post‐2015 expansion of *tmexCD‐toprJ* positive isolates co‐harboring *bla*_NDM_, *bla*_KPC_, or *mcr* (Figure 6). In contrast, hospital‐sector antimicrobial consumption trends in the EU/EEA showed more modest increases for tetracyclines, carbapenems, and colistins over the available period (Figure S10). These ecological data cannot establish direct causality at the isolate level. Nevertheless, the temporal association is consistent with the possibility that regional antimicrobial selection contributes to the maintenance and enrichment of dual‐resistance plasmids once they arise.

## 3 Discussion

This study provides a global genomic and plasmid level view of the convergence between the plasmid‐mediated tigecycline resistance gene cluster *tmexCD‐toprJ* and critical resistance determinants. Although *tmexCD‐toprJ* has been reported in multiple bacterial hosts and plasmid types, its global distribution and co‐occurrence with carbapenemase and colistin resistance genes have remained incompletely understood [1, 26]. By integrating public genomes with newly sequenced clinical isolates, we show that *tmexCD‐toprJ* is not confined to a single bacterial clone or plasmid lineage. Instead, its distribution across diverse bacterial sequence types, plasmid backbones, and ecological sources is more consistent with repeated plasmid‐associated acquisition and dissemination [27].

A key implication of our analysis is that *tmexCD‐toprJ* should not be viewed as an isolated tigecycline resistance determinant. In China, where the increase was most pronounced, a substantial proportion of post‐2015 *tmexCD‐toprJ*‐positive isolates also carried *bla*_NDM_, *bla*_KPC_, or *mcr*. This pattern is clinically important because it links tigecycline resistance with resistance to carbapenems or colistin, antibiotics that are often used as last‐line options for severe multidrug‐resistant bacterial infections [13, 28]. More importantly, the convergence of these determinants was not random. *tmexCD‐toprJ*‐*bla*_NDM_ plasmids were mainly associated with IncU/IncHI1B‐like backgrounds, whereas *tmexCD‐toprJ‐bla*_KPC_/*mcr* plasmids were more often linked to IncF related backbones. This replicon specific partitioning suggests that plasmid architecture helps shape which resistance modules can be assembled and maintained together.

Functional experiments supported the biological relevance of these genomic observations. Phenotypic testing confirmed that representative dual‐positive isolates expressed resistance to the corresponding antibiotic classes, indicating that co‐carriage of *tmexCD‐toprJ* with *bla*_NDM_, *bla*_KPC_, or *mcr* is functionally meaningful rather than merely a genomic association. Stability assays further showed that selected dual‐positive plasmids or co‐resistance determinants could be maintained during serial passage without antibiotic selection, suggesting that these resistance structures can persist when direct drug pressure fluctuates. Conjugation assays demonstrated that a subset of *tmexCD‐toprJ‐*carrying plasmids retained transferability, including plasmids co‐harboring *tmexCD‐toprJ* and *bla*_NDM_, indicating that combined resistance determinants can be disseminated in a single horizontal transfer event. These results do not imply that all dual‐positive plasmids are highly transmissible; rather, they show that certain plasmids can combine resistance expression, stable maintenance, and transfer potential, thereby creating resistance structures with clear clinical relevance. Therefore, surveillance of *tmexCD‐toprJ* should include not only detection of the gene cluster itself but also assessment of co‐localized carbapenemase and colistin resistance genes, plasmid backbones, and transfer potential.

The competition and antibiotic pressure experiments further illustrate how dual‐positive plasmids may persist or arise under selective conditions. In the same *E. coli* background, a *tmexCD‐toprJ‐bla*_NDM_ transconjugant outcompeted a *tmexCD‐toprJ*‐only transconjugant under tigecycline exposure despite having a lower intrinsic growth rate in monoculture. This suggests that competitive outcomes depend not only on basal growth kinetics but also on antibiotic conditioned selection and plasmid‐host interactions. In addition, the antibiotic‐pressure experiment showed that *tmexCD‐toprJ* can be mobilized into the *umuC*‐associated region of a *bla*_KPC_ plasmid in a representative strain carrying the two determinants on separate plasmids. This provides experimental support for the feasibility of *tmexCD‐toprJ‐bla*_KPC_ plasmid convergence under combined tigecycline and meropenem pressure. However, this experiment should be interpreted as evidence for one possible convergence route rather than proof that all naturally occurring dual‐positive plasmids arise through the same mechanism.

The ecological distribution of different resistance combinations points to a One Health dimension. *tmexCD‐toprJ*‐positive isolates carrying additional resistance determinants were detected across human, animal, and environmental sources, with distinct ecological distributions observed for different resistance gene combinations. Antibiotic consumption data also indicated substantial regional differences between China and EU/EEA countries. Similar associations between antimicrobial consumption and resistance emergence have been reported previously [29, 30]. Although our observations are consistent with the hypothesis that antimicrobial selection contributes to the accumulation of multidrug‐resistant plasmids, this relationship should be interpreted cautiously because the available genomic dataset may be influenced by surveillance intensity, sequencing practices, and sampling bias. Future studies integrating systematic surveillance with detailed antimicrobial usage data will be necessary to better quantify the contribution of antibiotic pressure to plasmid evolution and dissemination.

This study has several limitations. First, although prevalence‐based analyses were used to reduce bias from uneven sequencing intensity, public genomic databases remain subject to differences in sampling, sequencing, reporting, and metadata completeness across countries and years. Second, phenotypic and functional experiments were performed on representative strains available in our collection, and therefore may not capture the full diversity of global plasmid‐host backgrounds. Third, although the antibiotic pressure experiment showed that *tmexCD‐toprJ* can insert into an *umuC*‐associated region of a *bla*_KPC_ plasmid, targeted genetic manipulation of large multidrug resistance plasmids remains technically challenging. Additional engineered plasmid systems will be needed to define the precise recombination mechanism and determine how general this route is. Fourth, not all public genomes had long read sequencing data; therefore, plasmid reconstruction for some public isolates relied on contig prediction, replicon annotation, and structural inference.

In conclusion, our data showed that *tmexCD‐toprJ* converges with *bla*_NDM_, *bla*_KPC_, and *mcr* in distinct plasmid backgrounds, generating dual‐positive plasmids that can be phenotypically resistant, stably maintained, and in some cases transferable. These findings do not prove that *tmexCD‐toprJ* co‐carriage alone drives global dissemination. Rather, they show that plasmid convergence of tigecycline resistance with carbapenemase or colistin resistance determinants creates high‐risk resistance structures with clear clinical relevance. Integrated surveillance combining genomic, phenotypic, plasmid‐level, and One Health data will be essential for early detection and control of these emerging multidrug resistance plasmids.

## 4 Materials and Methods

### 4.1 Genome Collection and Dataset Construction

Publicly available *tmexCD‐toprJ*‐positive isolates were collected from multiple NCBI resources up to June 2024. Candidate isolates were first identified from the NCBI Pathogen Detection database (https://www.ncbi.nlm.nih.gov/pathogens/isolates), which contained antibiotic resistance annotations for 1 735 923 publicly available bacterial genomes. Isolates annotated as carrying a complete *tmexCD‐toprJ* gene cluster were selected as candidates. For candidates with available GenBank assembly accessions, the corresponding genome assemblies were downloaded directly. For candidates without GenBank assemblies but with linked Sequence Read Archive (SRA) accessions, raw sequencing reads were downloaded, assembled using Unicycler, and then subjected to the same downstream screening procedure.

For prevalence analysis, we used the subset of 1 401 543 isolates in the NCBI Pathogen Detection database with available collection year and geographic metadata. Associated metadata (isolation source, location, collection date, host) were concurrently downloaded. The presence of a complete *tmexCD‐toprJ* gene cluster was then verified using ABRicate against a custom database, requiring >85% nucleotide identity and coverage across all three genes. Potential duplicates were removed by metadata cross‐referencing and pairwise average nucleotide identity (ANI) analysis using FastANI, retaining only one representative from isolate pairs with ANI ≥99.99%. After quality control, confirmation of complete *tmexCD‐toprJ*, and de‐duplication, 1189 high quality, non‐redundant public genomes were retained.

In parallel, prospective multicenter clinical surveillance was conducted across 76 tertiary hospitals in China. 38 *tmexCD‐toprJ*‐positive clinical isolates were identified by polymerase chain reaction. Bacterial species were identified using MALDI‐TOF MS. Antimicrobial susceptibility testing was performed using standardized agar dilution methods according to CLSI guidelines, and minimum inhibitory concentrations (MICs) were determined for clinically relevant agents. *E. coli* ATCC 25922 and *P. aeruginosa* ATCC 27853 were used as quality‐control strains. These isolates were sequenced and were used for experimental validation.

The final combined dataset containing 1189 public genomes plus 38 in‐house sequenced clinical isolates (total *n* = 1227) was used for all analyses of the distribution and genetic characteristics of *tmexCD‐toprJ*. Detailed metadata are provided in Table S2.

### 4.2 Whole Genome Sequencing and Genome Annotation

Genomic DNA from 38 clinical isolates was extracted using the DP302 Kit (Tiangen Biotech). For short read sequencing, libraries were prepared using the NEBNext Ultra II DNA Library Prep Kit and sequenced on an Illumina NovaSeq 6000 platform to generate 150‐bp paired‐end reads. Long read libraries were prepared using the SMRTbell Express Template Prep Kit 2.0 and sequenced on a PacBio Sequel IIe system for HiFi reads. Raw Illumina reads were trimmed with Trimmomatic [31], while PacBio reads were processed via SMRT Link. Clinical isolates underwent hybrid assembly using Unicycler [32]. Assembly quality was assessed using QUAST [33] and CheckM [34].

All assembled genomes were annotated using Prokka [35] and Bakta [36]. Multi‐locus sequence typing (MLST) employed PubMLST [37]. Antibiotic resistance genes (ARGs) were identified using ABRicate with ResFinder [38] and CARD [39] (>85% identity, >85% coverage). The global spread of *tmexCD‐toprJ* includes both isolates carrying *tmexCD‐toprJ* alone and isolates co‐harboring additional resistance genes (co‐harboring ≥1 additional resistance gene, including *bla*_NDM_, *bla*_KPC_, or *mcr*). Plasmid replicon types were determined via PlasmidFinder [40]. Mobile genetic elements were characterized as follows: Insertion sequences using ISfinder [41]; integrative conjugative elements (ICEs) via ICEfinder [42].

To characterize plasmid‐encoded defense evasion modules, all genomes were screened against the Anti‐CRISPRdb and REBASE databases. Anti‐CRISPR proteins, restriction–modification systems, and other anti‐defense genes were identified using BLASTn with stringent thresholds (>85% nucleotide identity and >85% coverage). Hits were manually curated to remove spurious matches. The distribution and diversity of anti‐defense systems were quantified across *tmexCD‐toprJ*‐carrying plasmids, and entropy values were calculated to assess repertoire diversity.

To investigate the genetic relationships among *tmexCD‐toprJ*‐associated plasmids, plasmid pangenome analysis was performed using Roary [43]. Orthologous gene clusters present in >85% of plasmids and containing paralogs in <10% of plasmids were retained as core plasmid genes. Concatenated core genes were aligned with MAFFT [44]. Phylogenies were reconstructed via IQ‐TREE2. For public genomes with only short read sequencing data available, plasmid sequences were identified from contigs using PlasFlow [45] (threshold = 0.7). *tmexCD‐toprJ*‐harboring plasmids were annotated with Prokka/Bakta. Circular visualizations were generated using tvBOT [46]. The genetic context of *tmexCD‐toprJ* was manually annotated for MGE associations and visualized via comparative linear maps in genoPlotR [47].

### 4.3 Plasmid Copy Number Analysis

The plasmid copy number (PCN) was computed as the ratio of the mean coverage of plasmid contigs to that of the host chromosome. Further quality filters removed plasmids from assemblies with sequencing size <10 kb. All available assemblies annotated at the contig level. A power‐law model was employed to predict expected sequencing depth based on contig length, defined as predict_depth(length) = 10^3.43 × length^(−0.627), where length represents the contig length. This model was used to establish the expected relationship between contig length and sequencing depth [48]. To normalize sample specific variations in sequencing depth, an optimal adjustment factor (k) was computed for each sample. Using numerical optimization (the Brent method), k minimized the mean squared error between log‐transformed adjusted depths and log‐transformed predicted depths from the power‐law model. This adjustment aligned each sample's depth distribution with the model's expected length‐dependent pattern.

### 4.4 Stability Assays

Four clinical isolates were selected to evaluate the stability of *tmexCD‐toprJ*‐positive plasmids. These included C6947, in which *tmexCD‐toprJ* and *bla*_NDM_ were located on the same plasmid; C9296, in which *tmexCD‐toprJ* and *bla*_NDM_ coexisted in the same bacterial isolate but were located on different plasmids; C5625, in which *tmexCD‐toprJ* and *bla*_KPC_ were located on the same plasmid; and C4050, in which *tmexCD‐toprJ* and *bla*_KPC_ coexisted in the same bacterial isolate but were located on different plasmids.

Plasmid stability was assessed by continuous serial passage under non‐selective conditions. Briefly, overnight cultures were diluted 1:100 into fresh antibiotic‐free lysogeny broth and incubated at 37°C. This transfer was repeated daily for 15 days, corresponding to approximately 105 bacterial generations. At each passage point, cultures were diluted and plated onto antibiotic‐free solid medium. For each strain and condition, 96 randomly selected colonies were screened by polymerase chain reaction using primers targeting *tmexCD‐toprJ* and the corresponding co‐carried resistance determinant, *bla*_NDM_ or *bla*_KPC_. Experiments were performed with triplicate biological replicates. Plasmid maintenance frequencies were calculated as the proportion of screened colonies retaining the target resistance determinants at each time point.

### 4.5 Conjugation Assays

Conjugation experiments were performed using clinical isolates as donors and sodium azide resistant *E. coli* J53 as the recipient. The 38 in‐house isolates were tested as donor strains, including 8 strains carrying *tmexCD‐toprJ*‐only plasmids, 15 strains carrying *tmexCD‐toprJ* and *bla*_KPC_, *bla*_NDM_, or *mcr* on the same plasmid, and 15 strains carrying *tmexCD‐toprJ* and *bla*_KPC_, *bla*_NDM_, or mcr in the same bacterial isolate but on separate plasmids. Donor and recipient strains were cultured overnight in lysogeny broth (LB) at 37°C. Overnight cultures were diluted 1:100 into fresh LB and grown to mid‐exponential phase (OD600 ≈ 0.6). Cells were harvested by centrifugation, washed once with sterile phosphate‐buffered saline to remove residual antibiotics, and resuspended in fresh LB. Donor and recipient suspensions were mixed at a 1:1 ratio, diluted 1:100 into fresh LB, and incubated at 37°C for 16–18 h. After mating, serial dilutions were plated onto China Blue agar supplemented with sodium azide and the corresponding selective antibiotic or antibiotic combination to inhibit donor growth and select transconjugants. Candidate transconjugants were confirmed by polymerase chain reaction targeting *tmexCD‐toprJ*, *bla*_KPC_, *bla*_NDM_, or *mcr*, as appropriate, and by antimicrobial susceptibility testing. Conjugation frequencies were calculated as the number of confirmed transconjugants per recipient cell.

### 4.6 Competition Assays and Growth Curve Analysis

To evaluate the fitness effects associated with different *tmexCD‐toprJ*‐positive plasmid configurations, we performed both pairwise competition assays and growth curve analysis using Escherichia coli J53 transconjugants. For competition assays, two transconjugants were used: C6846T, carrying a *tmexCD‐toprJ*‐only plasmid, and C6356T, carrying a co‐resistant plasmid harboring both *tmexCD‐toprJ* and *bla*_NDM_. C6846T was labelled with the red fluorescent plasmid pWH1266‐mCherry, whereas C6356T was labelled with pWH1266‐GFP. Both strains were cultured overnight in lysogeny broth (LB) at 37°C with shaking, diluted 1:100 into fresh LB, and grown to exponential phase (OD600 ≈ 0.6). Equal volumes of the two cultures were then mixed at a 1:1 ratio and co‐cultured in fresh LB at 37°C with shaking. Samples were collected at 6, 12, and 24 h. Serial dilutions were plated onto LB agar plates with or without meropenem to enumerate competing populations, and the proportion of each strain was calculated based on colony‐forming units. To visualize population dynamics, aliquots collected at each time point were examined by fluorescence microscopy using red and green fluorescence channels.

Growth kinetics were measured for four strains: the recipient strain *E. coli* J53, C6846T carrying the *tmexCD‐toprJ*‐only plasmid, and C6356T and C6361T carrying *tmexCD‐toprJ‐bla*_NDM_ co‐resistance plasmids. Overnight cultures were diluted 1:100 into fresh LB and grown to exponential phase. Cultures were then adjusted to the same initial cell density and transferred into sterile 96‐well microplates. Bacterial growth was monitored at 37°C for 24 h by measuring OD600 at regular intervals using a microplate reader with continuous orbital shaking between measurements. Three independent biological replicates were performed for each strain.

### 4.7 Antibiotic‐Pressure Plasmid Convergence Experiment

To investigate whether antibiotic selective pressure promotes structural rearrangements involving the *tmexCD‐toprJ* gene cluster and the *umuC* recombination hotspot, strain C8182 was selected for further analysis. This strain carries *tmexCD‐toprJ* and *bla*_KPC_ on two separate plasmids, and the *bla*_KPC_‐carrying plasmid contains an intact *umuC* region. An overnight culture of C8182 was diluted 1:100 into fresh lysogeny broth (LB) supplemented with tigecycline and meropenem. Cultures were incubated at 37°C with shaking. Antibiotic concentrations were increased daily by 50% of the previous concentration over five consecutive passages, with daily 1:100 subculture into fresh selective medium. Following selection, two PCR strategies were applied to assess structural rearrangements. First, primers flanking the original *umuC* locus were used to determine whether the native configuration remained intact. Second, junction‐spanning primers were designed based on the predicted recombination model inferred from plasmid structure analysis and schematic reconstruction (Figure 6I) to detect potential insertion events. PCR products were compared between parental and evolved isolates to identify structural changes. Primer sequences are listed in Table S7.

### 4.8 Antibiotic Consumption Data

The dataset on inpatient antibiotic use in China is derived from hospitals participating in the Centre for Antibacterial Surveillance (CAS), details quarterly aggregate data on inpatient antibiotic use from January 2015 to September 2024. It encompassed over 5000 hospitals across all 31 provinces of mainland China. In Europe, antimicrobial consumption data is mainly collected and reported via the European Surveillance System in accordance with the ESAC‐Net reporting protocol. The data involves 25 EU Member States and 2 EEA countries (Iceland and Norway) that participate in disease surveillance. For the classification and analysis of antimicrobials, the Anatomical Therapeutic Chemical (ATC) classification system is utilized, with the latest ATC/DDD (Defined Daily Dose) index applied to consumption data throughout all years. Focusing on the hospital sectors within the EU/EEA and individual countries, antibiotic consumption is presented as the number of DDDs per 1000 inhabitants per day.

### 4.9 Statistical Analysis

Statistical analyses were implemented in R software. In terms of the number of insertion sequences per genome, the Mann–Whitney U test was applied to make a comparison between *tmexCD‐toprJ*‐only strains and dual‐positive strains, so as to examine whether there was a significant difference in the quantity of IS per genome between these two types of strains. For the temporal distribution of samples, the Mann‐Whitney U test was used to compare the collection years between the human derived group and the animal/environment‐derived group, aiming to identify potential differences in the timing of occurrence between the two sources. All statistical tests were two‐tailed, and a *p*‐value less than 0.05 was considered statistically significant. Data visualization was generated using the ggplot2 [49] package in R to visually illustrate the temporal distribution characteristics of the two groups.

## Author Contributions

H.W. conceived and designed the study. Q.W., X.W. collected the bacterial isolates and sequenced them. K.S. and Q.D. conducted microbial susceptibility testing and stability assays on the bacterial strains. G.X. has conducted monitoring on the usage of antibiotics in hospitals in China over time. Bioinformatics analyses were performed by K.S. and M.W. K.S. wrote the draft. H.W. and M.W. performed revisions. During this process, H.W., M.W., M.C., B.L., and R.W. provided a lot of guidance for background research, article writing, image production, and other items. K.S. and M.W. have verified the underlying data. The authors have read and approved the final manuscript.

## Funding

This work was supported by the National Key Research & Development Program of China (Grant No. 2023YFC2307100 to H.W.). National Natural Science Foundation of China (Grant No. 32141001 to H.W.).

## Conflicts of Interest

The authors declare no conflicts of interest.

## Supporting information




**Supporting File 1**: advs77169‐sup‐0001‐SuppMat.docx.

**Supporting File 2**: advs77169‐sup‐0002‐S1.xlsx.

**Supporting File 3**: advs77169‐sup‐0003‐S2.xlsx.

**Supporting File 4**: advs77169‐sup‐0004‐S3.xlsx.

**Supporting File 5**: advs77169‐sup‐0005‐S4.xlsx

**Supporting File 6**: advs77169‐sup‐0006‐S5.xlsx.

**Supporting File 7**: advs77169‐sup‐0007‐S6.xlsx.

**Supporting File 8**: advs77169‐sup‐0008‐S7.xlsx.

## Data Availability

All new data used for analysis have been submitted to NCBI, with BioSample accessions ranging from SAMN62200489 to SAMN62200526.
