# Supplementary material for: Convergence of Plasmid‐Mediated tmexCD‐toprJ With Critical Resistance Genes Fuels Spread of Multidrug Resistant Bacteria
Source: Adv Sci (Weinh). 2026 Aug 20;13(62):e77169. doi: 10.1002/advs.77169 (PMC13491359; doi:10.1002/advs.77169)
Supplement: Supplementary file 1 [file ADVS-13-e77169-s003.docx]

Supplementary Materials for

**Convergence of Plasmid-Mediated tmexCD-toprJ with Critical Resistance Genes Fuels Spread of Multidrug-Resistant Bacteria**

KaiWen Song *et al.*

*Corresponding author. Email: Hui Wang, whuibj@163.com, wanghui@pkuph.edu.cn

**This PDF file includes:**

Figures. S1 to S11

**Other Supplementary Materials for this manuscript include the following:**

Tables S1 to S7

Figure S1. Distribution of *tmexCD-toprJ*-positive strains. (A)Worldwide geographic distribution of bacterial strains harboring *tmexCD-toprJ* genes, with color gradients indicating regional prevalence. (B) Global geographic distribution of bacterial strains harboring *tmexCD-toprJ* genes and at least one of the resistance genes (*bla*_NDM_, *bla*_KPC_, *mcr*). (C) Temporal trends in the number of bacterial strains harboring *tmexCD-toprJ* genes combined with other resistance genes (*bla*_NDM_, *bla*_KPC_, *mcr*). Lines represent strains where *tmexCD-toprJ* and resistance genes are localized on same plasmids, while bars show strains with co-occurring *tmexCD-toprJ* and resistance genes in the same bacterial isolate. (D) Proportions of the top six bacterial genera among strains harboring *tmexCD-toprJ* genes in co-occurrence with other resistance genes (*bla*_NDM_, *bla*_KPC_, *mcr*).

Figure S2. Temporal prevalence of *tmexCD-toprJ*-positive isolates across different countries/regions. (A) Line graph showing the temporal trends of *tmexCD-toprJ* prevalence (%) in China compared with all other countries combined. Data are derived from the NCBI Pathogen Detection database. (B) Bubble plot showing the distribution of *tmexCD-toprJ*-carrying isolates in major countries/regions. Bubble size represents the absolute number of *tmexCD-toprJ*-positive isolates for each country/region per year, and bubble color indicates the prevalence (%) of *tmexCD-toprJ*-positive isolates.

Figure S3. Genetic context and *tmexCD-toprJ* gene clusters. (A) Phylogenetic reconstruction of *tmexCD-toprJ*-positive plasmids. Phylogenetic tree reconstructed using MAFFT for multiple sequence alignment, IQ-TREE with the GTR+G model, and 1,000 bootstrap replicates for branch support. (B) Structural organization of six major *tmexCD-toprJ* gene variants, highlighting flanking mobile genetic elements with pink shading indicating putative horizontal transfer modules.

**Figure S4. Anti-defense systems in resistance plasmids.** (A) Anti-defense gene count distribution stratified by resistance gene. (B) Shannon entropy analysis of anti-defense gene diversity, with higher entropy values indicating broader gene-type distributions within plasmid groups. Anti-CRISPR proteins were detected using Anti-CRISPRdb, while restriction-modification systems and related modules were identified using REBASE. Each genome was screened with thresholds of >85% identity and coverage.

**Figure S5. Replication origins and size variation in *tmexCD-toprJ*-positive plasmids.** Solid circles indicate plasmids with only one replication origin, while open circles represent multi-replicon plasmids containing two or more distinct replication origins. The y-axis denotes the average genomic size of each plasmid group, and the x-axis displays the categorization of plasmids based on their replication systems. Each edge connecting separate nodes corresponds to mosaic hybrid plasmids that simultaneously possess different types of replicons.

**Figure S6. Heatmap of similarity among IncU plasmids.** Heatmap showing the similarity of IncU plasmids, with the color gradient indicating similarity levels: green represents low similarity and red represents high similarity. The x-axis denotes plasmid types, while the y-axis indicates types of carried resistance genes. Plasmid similarity network. Similarity was defined based on the number of shared genes between plasmids.

**Figure S7. Heatmap of similarity among repB plasmids.** Heatmap showing the similarity of repB plasmids, with the color gradient indicating similarity levels: green represents low similarity and red represents high similarity. The x-axis denotes plasmid types, while the y-axis indicates types of carried resistance genes.

**Figure S8. Heatmap of plasmid-borne resistance gene profiles across different isolates.** This heatmap visualizes the distribution of resistance gene combinations in plasmids carrying *umuC* alleles, categorized by genus (rows) and resistance gene type (columns).

**Figure S9. Trends in antibiotic consumption in the EU/EEA in the hospital sector from 2013 to 2022.** Line graph showing the annual consumption of four classes of antibiotics in the EU/EEA in the hospital sector over the decade, measured in defined daily doses (DDD) per 1,000 inhabitants per day. The antibiotics included are tetracyclines (J01A), carbapenems (J01DH), and colistins (J01XB). The x-axis denotes the years (2013-2022), and the y-axis represents the consumption in DDD per 1,000 inhabitants per day.

**Figure S10. Temporal distribution and comparative analysis of *tmexCD-toprJ-bla*_NDM_, *tmexCD-toprJ-bla*_KPC_, and *tmexCD-toprJ-mcr* across human and animal/environmental sources.** (A) Stacked histograms showing the annual distribution of samples for each gene type, with human sources in orange and animal/environmental sources in blue. The y-axis represents sample count, and data are stratified by gene type in separate panels. (B) Boxplots comparing the distribution of collection years between human and animal/environmental sources for each gene type. Boxes represent interquartile ranges with median lines; points show individual data points. Statistical significance of differences (Mann-Whitney U test) is indicated by asterisks.

**Figure S11. Relationship between plasmid size and sequencing depth.** Scatter plots showing the relationship between plasmid size (X-axis, log_10_ scale, in base pairs) and sequencing depth (Y-axis, log_10_ scale) for plasmid contigs. Each point represents an individual plasmid, with colored points indicating plasmids carrying specific resistance genes. Plasmids from the same strain are connected by dashed gray lines. The solid red line denotes the theoretical sequencing depth predicted by the power-law model (depth = 10^3.43^ × length^-0.627^), where points above the line indicate higher-than-expected depth (relative to their length) and points below indicate lower-than-expected depth.

Tables S1–S3.

**Table S1**. Metadata of all *tmexCD-toprJ*–carrying isolates analyzed in this study. This table includes two subsets:

- lab: Isolates collected from the national CRE surveillance program in China.
- public: Isolates retrieved from public databases.

**Table S2**. Isolates carrying plasmids co-harboring *tmexCD-toprJ* and at least one of *bla*_NDM_, *bla*_KPC_, or *mcr*.

**Table S3**. Isolates co-carrying *tmexCD-toprJ* and at least one of *bla*_NDM_, *bla*_KPC_, or *mcr* within the same strain.

**Table S4.** Positive rate of isolates carrying *tmexCD-toprJ in* different country.

**Table S5.** Donor strains, recipient strain, and transconjugants generated in conjugation assays.

**Table S6.** Fluorescent reporter strains used for competition assays.

**Table S7.** Primers used for PCR and junction detection in this study.
